# A 5-Year Update on the Clinical Development of Cancer Cell-Based Vaccines for Glioblastoma Multiforme

**DOI:** 10.3390/ph18030376

**Published:** 2025-03-06

**Authors:** Almohanad A. Alkayyal, Ahmad Bakur Mahmoud

**Affiliations:** 1 Department of Medical Laboratory Technology, Faculty of Applied Medical Sciences, University of Tabuk, Tabuk 71491, Saudi Arabia; 2 College of Applied Medical Sciences, Taibah University, Madinah 41477, Saudi Arabia; 3 Health and Life Research Center, Taibah University, Madinah 42353, Saudi Arabia

**Keywords:** GBM, cancer cell vaccine, cell lysate, DC vaccine, immunotherapy

## Abstract

Glioblastoma multiforme (GBM) is considered one of the most aggressive forms of brain cancer with a 15-month median survival, despite advancements in surgery, radiotherapy, and chemotherapy. The immune-suppressed tumor microenvironment and the blood–brain barrier are major contributors to its poor prognosis and treatment resistance. In the last decade, significant progress has been made in developing cell-based vaccines to boost immune responses against GBM. This review provides an extensive update on recent clinical trials involving various cancer cell vaccines, including ICT-107, the α-type-1 DC vaccine, and others. Although these trials have demonstrated potential improvements in progression-free survival (PFS) and overall survival (OS), the diverse and immune-suppressed nature of GBM poses challenges for consistent therapeutic success. We discuss the details of these trials along with the potential mechanism of vaccine efficacy and immune activations. The findings of these trials highlight the significance of a personalized immunotherapy approach and suggest that patient stratification could significantly advance the clinical management of GBM.

## 1. Introduction

Glioblastoma (GBM) is classified as one of the most aggressive types of central nervous system (CNS) cancers [1]. The current standard treatment for GBM involves maximal safe surgical resection, combined with adjuvant therapies such as radiotherapy and/or temozolomide (TMZ) chemotherapy [2,3]. However, there has been minimal change in these treatments for nearly two decades, resulting in a median overall survival (OS) of approximately 15 months from diagnosis for patients [2,3]. Regrettably, a high percentage of GBM patients face tumor recurrence [4,5]. This high recurrence rate is often attributed to quiescent tumor-seeding cells situated in unresectable regions far from the original tumor site early in the tumor’s development, or to infiltrated tumor cells at the boundary between the tumor and normal brain tissue [6,7]. According to the latest classification by the World Health Organization (WHO), adult malignant gliomas are divided into specific categories, and it is crucial to note that low-grade gliomas can progress to more malignant and lethal higher-grade gliomas [8]. In cases where treatment is inadequate, residual tumor cells may create a virtual connection with nearby normal cells, enabling them to transfer genetic material and abnormal proteins, thus altering the phenotype of these normal cells [9,10,11]. This transfer can occur through the direct formation of gap junctions or the release of tumor-derived extracellular vesicles, aiding damaged tumor cells’ survival [10,11]. To better manage GBM, additional therapies are required to diminish the unseen tumor burden and impede tumor progression. One promising strategy is to activate the body’s immune response through immunotherapy, which has long been considered a potential approach for enhancing GBM outcomes [12]. Integrating immunotherapy into the current standard of care for GBM could represent a crucial next step in enhancing the effectiveness of treatment for patients [12].

Immunotherapy has revolutionized cancer treatment over the past decade, encompassing a range of modalities, including adoptive cell transfer immunotherapy (such as chimeric antigen receptor T cells (CAR-Ts) and T cell receptor-engineered T cells (TCR-Ts)), passive immunotherapy with antibodies, active specific immunotherapy like vaccines, modulatory immunotherapy involving checkpoint inhibitors and cytokines, and immunogenic cell death (ICD) therapy such as oncolytic virus therapy [13,14]. While some therapies, like Immunogenic Cell Death inducers (ICDs), are designed to induce a generalized immune response against tumors without customization to individual patient characteristics, others are specifically tailored to target antigens present on a patient’s tumor, thereby offering more personalized treatment [15,16,17]. The most common personalized approaches, including cancer cell-based vaccines and autologous dendritic cell (DC) vaccines, consider both the specific tumor antigens and the patient’s unique immune profile to optimize the immune response [18,19,20,21]. Despite the evolving classification and terminology of these therapies, their core objective remains consistent: to harness the body’s immune system to effectively target and eliminate cancer.

However, the treatment of glioblastoma (GBM) remains particularly challenging due to its heterogeneous and immunosuppressive microenvironment. GBM tumors are characterized by genetic and phenotypic diversity, which allows them to develop resistance to conventional therapies [22,23]. The blood–brain barrier (BBB) further complicates treatment by limiting the delivery of therapeutic agents to the tumor site [23,24]. Additionally, GBM actively suppresses the immune system through multiple mechanisms, such as the secretion of immunosuppressive cytokines, recruitment of regulatory T cells (Tregs), and expression of immune checkpoint molecules like PD-L1 [24,25]. These factors collectively contribute to the poor prognosis associated with GBM and underscore the urgent need for innovative therapeutic strategies.

Within the GBM microenvironment, tumor-associated macrophages (TAMs) are among the most abundant immune cell types and are often polarized towards an M2 phenotype, which supports tumor growth and suppresses immune responses [24,26]. M2-TAMs secrete anti-inflammatory cytokines like IL-10 and TGF-β, which inhibit cytotoxic T cell activity and promote the recruitment of Tregs [26]. Regulatory T cells (Tregs) further suppress the activity of effector T cells and other immune cells that could potentially target and destroy tumor cells [16,26,27,28]. GBM tumors also release chemokines such as CCL2 and CCL22, which attract Tregs to the tumor site [29,30]. Myeloid-derived suppressor cells (MDSCs) are another critical component of this immunosuppressive environment, inhibiting T cell activation and proliferation through the production of reactive oxygen species (ROS), nitric oxide (NO), and immunosuppressive cytokines [31,32,33,34]. These intricate immune evasion mechanisms highlight the complexity of GBM and the need for multifaceted immunotherapeutic approaches to improve treatment outcomes.

In this review, we provide a comprehensive update on the clinical development of cell-based vaccines for glioblastoma multiforme (GBM) over the past five years. We delve into the clinical trials that have shaped our current understanding of their efficacy. Additionally, we explore the ongoing challenges faced in translating these therapies into effective clinical treatments and discuss future perspectives for enhancing the success of cell-based vaccines in GBM immunotherapy.

### 1.1. GBM6-AD—A Phase I Trial

This study examined the safety and immune-boosting properties of a neoadjuvant vaccination approach using a GBM6-AD vaccine, a combination of tumor-cell lysate and poly-ICLC, in patients diagnosed with low-grade gliomas (LGGs) [35]. Seventeen participants were recruited and randomly assigned to receive the vaccine either before or after surgical standard-of-care intervention [35].

The preparation of the GBM6-AD tumor-cell lysate vaccine involves several critical steps designed to prime the immune system to recognize and attack glioma cells effectively [35]. The process involves the collection and culturing of tumor cells from the GBM6 cell line, known for expressing antigens commonly found in glioma cells [35]. Once sufficient cells are cultured, they undergo lysis to release their intracellular contents, including tumor-associated antigens (TAAs), which are crucial for stimulating an immune response [35]. To enhance the immune response, the tumor-cell lysate is combined with poly-ICLC, a synthetic double-stranded RNA that acts as a potent immune adjuvant [35]. Poly-ICLC activates pattern recognition receptors like toll-like receptor 3 (TLR3) on dendritic cells, leading to the production of type-1 interferons and other cytokines that promote a Th1-type immune response [35,36]. The vaccine is then carefully aliquoted into doses for intradermal administration, either before surgery (neoadjuvant) or after surgery [35]. The intradermal route is chosen because it effectively delivers the vaccine to immune cells in the skin, particularly dendritic cells, which then present the antigens to T cells in the lymph nodes [19,37,38,39]. After vaccination, patients are monitored for immune responses, including cytokine levels and the presence of activated T cells, both in the blood and within the tumor microenvironment (TME) [35]. This preparation process ensures that the vaccine delivers a consistent and effective dose of tumor antigens and immune stimulation, aiming to enhance anti-tumor immunity in patients with LGG.

The results demonstrated that the neoadjuvant vaccination was well tolerated and led to a significant increase in type-1 cytokines and chemokines, as well as heightened activation of CD8^+^ T cells in the peripheral blood [35]. Moreover, the immunological analysis indicated that the vaccine successfully primed the immune system, resulting in the migration of vaccine-reactive CD8^+^ T cell clones into the tumor microenvironment (TME) [35]. Single-cell RNA sequencing further confirmed the presence of these activated T cells within the TME, suggesting that the neoadjuvant vaccine could potentially overcome some of the immunosuppressive barriers commonly observed in gliomas [35]. However, despite these encouraging immunological findings, the clinical outcomes revealed a median progression-free survival (PFS) of 11 months and a median event-free survival (EFS) of 23.7 months, underscoring the need for further optimization of the vaccine regimen [35].

These results highlighted the potential of neoadjuvant vaccination strategies in boosting anti-tumor immunity in LGG patients. However, the study also underscores the necessity for further enhancements to improve the clinical effectiveness of such vaccines, particularly by integrating approaches that can more effectively counteract the immunosuppressive tumor microenvironment. Future investigations should concentrate on refining the combination of tumor-cell lysate vaccines with other immunotherapeutic strategies to achieve improved clinical outcomes for patients diagnosed with low-grade gliomas.

### 1.2. Autologous Dendritic Cell Vaccine Pulsed with Allogeneic Stem-like Cell Line Lysate—Phase I Trial

In a Phase I clinical trial, a dendritic cell (DC) vaccine was evaluated in patients with newly diagnosed or recurrent glioblastoma (GBM) [40]. The trial involved 36 patients, 11 with newly diagnosed GBM and 25 with recurrent GBM, to assess the vaccine’s safety, tolerability, and immunogenicity [40]. The production of the autologous dendritic cell (DC) vaccine in this study involved a complex process aimed at leveraging the patient’s immune system to combat glioblastoma [40]. The process commenced with the collection of peripheral blood mononuclear cells (PBMCs) via leukapheresis, which isolates the necessary monocytes [40]. These monocytes were subsequently cultured in a growth medium supplemented with granulocyte-macrophage colony-stimulating factor (GM-CSF) and interleukin-4 (IL-4) for four days, facilitating their differentiation into immature dendritic cells [40]. The dendritic cells were pulsed with the tumor lysate for 20 to 24 h [40]. This lysate contained a diverse range of tumor-associated antigens (TAAs), enabling the dendritic cells to effectively process and present these antigens, priming them for immune activation upon reintroduction into the patient [40]. To further mature the dendritic cells and enhance their antigen-presenting capabilities, they were pulsed with interferon-gamma (IFN-γ) and lipopolysaccharide (LPS). Stringent quality control measures ensured that the dendritic cells were appropriately matured, characterized by the expression of key markers such as CD40, CD80, CD83, CD86, HLA-ABC, HLA-DR, and CCR7, with low levels of CD14 [40]. The final product was divided into doses each containing approximately 10 million dendritic cells, which were cryopreserved until required [40]. The vaccine was administered via intradermal injection in the axillary region, with patients receiving the vaccine weekly for four weeks during the induction phase, followed by maintenance doses every eight weeks [40]. This regimen was formulated to optimize the immune response and maintain it over time, with the goal of effectively targeting glioblastoma cells and overcoming the tumor’s resistance to traditional therapies [40].

The trial yielded promising clinical outcomes, especially for newly diagnosed GBM patients. The median progression-free survival (PFS) was 8.75 months, and the median overall survival (OS) reached 20.26 months, representing a notable improvement given the typically poor prognosis of GBM [40]. In the recurrent GBM cohort, the median PFS was 3.22 months, with a median OS of 11.97 months. These results suggest a potential survival benefit, particularly for newly diagnosed patients [40]. Additionally, the vaccine demonstrated the ability to induce a cytotoxic T cell response in 36% of assessable patients, as evidenced by increased IFN-γ production in an ELISpot assay, highlighting its potential to stimulate a targeted immune response against glioblastoma cells [40].

### 1.3. An Autologous Tumor Lysate-Pulsed Dendritic Cell (DC) Vaccine in Combination with Gliadel Wafer—A Phase I Trial

In this Phase I trial, the safety, immune response, and potential synergy of combining Gliadel Wafer placement with autologous tumor lysate-pulsed dendritic cell (DC) vaccination in patients with malignant glioma, including glioblastoma (GBM), anaplastic astrocytoma (AA), and anaplastic oligodendroglioma (AO) was examined [41]. After maximal tumor resection, Gliadel Wafers containing carmustine (BCNU) were placed into the resection cavity to deliver localized chemotherapy [41]. Subsequently, patients received three rounds of autologous tumor lysate-pulsed DC vaccines at two-week intervals. The primary endpoints were safety, immune response, and overall survival (OS) and progression-free survival (PFS). The median OS for all GBM patients was 16.9 months, with a median PFS of 3.6 months [41].

The process of preparing the dendritic cell (DC) vaccine in the trial involved several important steps. After the surgical removal of the tumor, tumor samples were processed to extract tumor lysate [41]. Peripheral blood mononuclear cells (PBMCs) were obtained from patients through leukapheresis. These PBMCs were then cultured for seven days with recombinant human granulocyte macrophage colony-stimulating factor (GM-CSF) and interleukin-4 (IL-4) in complete medium to generate autologous DCs [41]. The day before each vaccination, the DC cultures containing 10^7^ to 10^8^ cells were washed and incubated overnight with autologous tumor lysate to facilitate their interaction [41]. The final vaccine, comprising tumor-specific, lysate-pulsed DCs, was administered to patients intradermally in the axillary region at 2-week intervals for three doses [41]. The tumor antigens presented by the pulsed dendritic cells stimulate the activation of cytotoxic T cells, which then target and eliminate any remaining tumor cells [41]. The inclusion of Gliadel Wafers contributes to reducing the local tumor burden, creating a synergistic effect with the systemic immune response generated by the dendritic cell vaccine [41]. This combined approach seeks to enhance immune recognition and precise elimination of tumor cells, while the localized chemotherapy provides supplementary anti-tumor activity to prevent recurrence [41].

The findings indicated that the combination of Gliadel Wafers and DC vaccination was well tolerated, with no severe adverse events reported [41]. Among 20 patients, immunogenicity data showed that 25% (5 of 20) responded to the DC vaccine, as evidenced by an increase in IFN-γ levels post-vaccination [41]. Notably, 80% of responders had recurrent GBM. Despite these modest immune responses, the overall clinical outcomes were similar to those observed in previous studies of DC vaccination alone, suggesting that adding Gliadel Wafer therapy may not significantly enhance vaccine efficacy [41]. However, patients who received adjuvant chemotherapy following DC vaccination tended to have slightly better survival outcomes, highlighting the potential benefit of combining immunotherapy with standard care [41].

In summary, this study demonstrates the safety of combining Gliadel Wafer implantation with autologous DC vaccination for malignant glioma. However, the modest immune response and survival benefit observed suggest the need for further optimization. Future studies should explore alternative combinations, such as checkpoint inhibitors or metabolic modulators, and conduct randomized controlled trials with rigorous molecular characterization of tumors to better understand the potential synergistic effects of this combined therapeutic approach.

### 1.4. ICT-107 Dendritic Cell Based Vaccine—A Phase II Trial

This Phase II randomized, double-blind, placebo-controlled trial evaluated the ICT-107 dendritic cell vaccine in newly diagnosed glioblastoma (GBM) patients [42]. ICT-107 targets six GBM-associated antigens, aiming to improve progression-free survival (PFS) and overall survival (OS) [42]. The study enrolled 124 patients, randomized 2:1 to receive either ICT-107 or a placebo after standard radiotherapy and temozolomide (TMZ) treatment. Although the primary endpoint of median OS did not show statistically significant improvement, the vaccine demonstrated a significant enhancement in PFS, particularly in HLA-A2+ patients with MGMT promoter methylation, who exhibited the most substantial clinical benefits [42].

The mechanism of action for ICT-107 involves pulsing autologous dendritic cells with peptide epitopes from six antigens—MAGE-1, HER-2, AIM-2, TRP-2, gp100, and IL13Ra2—targeting both tumor cells and cancer stem cells to reduce tumor heterogeneity and limit tumor escape [42]. The vaccine was well tolerated, with a safety profile comparable to the placebo group [42]. The trial also indicated that ICT-107 may be particularly beneficial in specific patient subgroups, such as HLA-A2+ patients with MGMT promoter methylation, who showed longer PFS and improved OS [42].

These findings suggest that while ICT-107 did not significantly improve OS in the overall study population, it holds promise for certain subgroups, underscoring the importance of personalized immunotherapy in GBM treatment [42]. Future research should focus on refining patient selection criteria and exploring combination therapies to maximize the therapeutic potential of ICT-107 and similar dendritic cell vaccines in the clinical management of GBM.

### 1.5. AV-GBM-1 (A Tumor-Initiating Cell Targeted Dendritic Cell Vaccine)—Phase II

This Phase II clinical study assessed the safety, feasibility, and effectiveness of AV-GBM-1, an autologous dendritic cell (DC) vaccine tailored to target tumor-initiating cells (TICs) in individuals diagnosed with newly developed glioblastoma (GBM) [28]. The primary aim of AV-GBM-1 is to activate the patient’s immune system to identify and combat TICs, which are known to contribute significantly to tumor recurrence and resistance to standard treatments like radiation and temozolomide (TMZ) [28]. Initially, 63 patients were enrolled, with 60 going through leukapheresis for vaccine production [28]. The vaccine was successfully manufactured for 57 patients, demonstrating a 97% success rate in production, indicating the feasibility and scalability of this approach in a clinical setting [28]. The vaccine was administered alongside standard-of-care treatments (RT/TMZ) in a schedule designed to enhance immune activation without disrupting the established therapeutic regimen [28].

The preparation of AV-GBM-1 involved several crucial steps to customize the vaccine for each patient [28]. Firstly, peripheral blood mononuclear cells (PBMCs) were collected via leukapheresis, isolating the immune cells necessary for generating dendritic cells [28]. These dendritic cells were then cultured and differentiated in a laboratory using growth factors such as granulocyte-macrophage colony-stimulating factor (GM-CSF) and interleukin-4 (IL-4) [28]. Once matured, the dendritic cells were loaded with a lysate from the patient’s own tumor-initiating cells, obtained from the glioblastoma tissue during surgical resection [28]. This lysate contains TIC-specific antigens, known for their resistance to standard therapies and their significant role in tumor recurrence [28]. After dendritic cells were loaded with the tumor lysate, they were administered to the patient via subcutaneous injection in a series of scheduled doses aimed at stimulating a potent immune response over time [28].

The clinical outcomes of the trial indicated that AV-GBM-1 holds potential to enhance survival rates in GBM patients, particularly in terms of progression-free survival (PFS) [28]. The median PFS stood at 10.4 months, a significant improvement compared to the typical 6.9 months seen in patients receiving standard care alone [28]. The median overall survival (OS) was 16.0 months, slightly surpassing the OS benchmark of 14.6 months from other GBM trials [28]. These findings suggest that the addition of the AV-GBM-1 vaccine may offer a modest survival advantage when combined with standard treatment protocols [28]. Importantly, the vaccine was generally well-tolerated, with most adverse events being mild, such as injection site reactions (15.8%) and flu-like symptoms (10.5%) [28]. However, the study reported a higher-than-expected rate of seizures (33%), which may be attributed to the inflammatory immune response triggered by the vaccine within the brain. This underscores the need for further safety assessments in future trials [28]. Overall, these results support the potential of AV-GBM-1 as a promising therapeutic approach for GBM, but larger randomized studies are necessary to confirm these findings and further refine the safety profile of the vaccine [28].

### 1.6. The α-Type-1 Dendritic Cell (DC) Vaccine—A Phase II Trial

The α-type-1 dendritic cell (DC) vaccine is an advanced form of immunotherapy intended to treat aggressive tumors such as glioblastoma multiforme (GBM) by triggering a targeted immune response [43]. This vaccine is developed by extracting dendritic cells from the patient and then converting them to an α-type-1 state to enhance their capacity to generate interleukin-12 (IL-12) [43]. IL-12 plays a vital role in stimulating a strong Th1-type immune response, which activates cytotoxic T lymphocytes (CTLs) to attack and eliminate tumor cells [43]. The dendritic cells are loaded with synthetic peptides derived from glioma-specific antigens, ensuring that the immune response specifically targets the tumor cells when the vaccine is administered back to the patient [43].

From a mechanistic standpoint, the α-type-1 DC vaccine functions by displaying these tumor-specific antigens on the surface of the dendritic cells through major histocompatibility complex (MHC) molecules [44]. This presentation triggers CTLs to identify and annihilate tumor cells presenting these antigens. The design of the vaccine also involves a positive feedback loop between IL-12 and interferon-gamma (IFN-γ), which intensifies and maintains the immune response [45]. This results not only in the immediate activation of CTLs but also in the promotion of long-term immune memory, which is crucial for preventing tumor recurrence.

The vaccine underwent evaluation in a Phase II clinical trial involving 16 patients diagnosed with high-grade gliomas, including GBM [43]. These patients were newly diagnosed and had received standard treatments, including maximal safe surgical resection, followed by radiation therapy and temozolomide (TMZ) chemotherapy [43]. The trial sought to examine the safety and effectiveness of the vaccine in enhancing survival outcomes when combined with standard therapy [43]. In this trial, each patient received the α-type-1 DC vaccine following completion of their initial standard treatment regimen. The vaccine was administered intradermally, and the DCs were tailored to be polarized to an α-type-1 state, enhancing their capability to generate interleukin-12 (IL-12) [43]. This cytokine is crucial for initiating a robust immune response against the tumor [43]. The dendritic cells were loaded with a mixture of synthetic peptides derived from glioma-specific antigens, ensuring that the immune response was highly focused on the tumor cells. The trial yielded promising results. After a six-year follow-up period, 5 out of the 16 patients were still alive, with 2 patients remaining free from relapse [43]. The median relapse-free survival (RFS) was 11 months, indicating that half of the patients experienced no tumor progression for nearly a year following treatment. The median overall survival (OS) was 19 months, signifying that half of the patients survived for over a year and a half after diagnosis [43]. These survival benefits are noteworthy, particularly considering the typically poor prognosis associated with GBM [43]. The trial also demonstrated that the vaccine was well-tolerated, with no significant adverse events directly linked to the vaccine, further supporting its potential as a viable treatment option in this challenging patient population.

### 1.7. CombiG-vax—A Phase II Trial

The CombiG-vax Phase II trial demonstrated encouraging outcomes in the treatment of glioblastoma using a dendritic cell (DC)-based vaccine [46]. Conducted as a monocentric study, the trial aimed to evaluate the safety, immunogenicity, and clinical efficacy of the CombiG-vax vaccine in combination with standard-of-care treatments, including surgical resection and temozolomide (TMZ) [46]. The trial enrolled 40 patients with newly diagnosed glioblastoma who received intradermal vaccine injections after completing standard chemoradiotherapy. The results indicated a median progression-free survival (PFS) of 11.3 months from leukapheresis and 12.2 months from surgery, with a median overall survival (OS) of 23.4 months [46]. Notably, the vaccine was well tolerated, with no severe treatment-related adverse events, emphasizing its safety profile. Immune monitoring revealed robust antigen-specific immune responses in a subset of patients, correlating with the improved clinical outcomes of these patients [46].

The vaccine preparation begins with isolating PBMCs from patients through leukapheresis, which were then cultured with GM-CSF and IL-4 to differentiate them into immature dendritic cells [46]. These cells were subsequently pulsed with autologous tumor-associated antigens (TAAs), derived from processed tumor lysates at the dose of 100 mg/mL [46]. The matured DCs, further stimulated using a cytokine maturation cocktail (IL-1β, TNF-α, IL-6, and prostaglandin E2), express high levels of co-stimulatory molecules (CD80, CD86) and MHC complexes, critical for antigen presentation. The vaccine’s immune adjuvant, keyhole limpet hemocyanin (KLH), was used to enhance its immunogenicity, ensuring a robust and durable immune response. This preparation ensures the vaccine’s capacity to effectively prime the immune system against GBM [46].

Mechanistically, CombiG-Vax relies on the unique antigen-presenting capabilities of dendritic cells to initiate and sustain anti-tumor immunity. Once administered, the vaccine delivers TAAs to cytotoxic T lymphocytes (CTLs) through MHC molecules, enabling these immune cells to recognize and eliminate GBM cells expressing the presented antigens. By targeting a broad range of TAAs, CombiG-Vax addresses the tumor heterogeneity characteristic of GBM, which often complicates treatment [46]. The vaccine’s mechanism also involves modulating the tumor microenvironment by reducing the number of regulatory T cells (Tregs) and increasing cytotoxic CD8^+^ T cells, shifting the balance toward an anti-tumor immune state [46].

The trial employed a structured administration schedule to optimize therapeutic outcomes. In the induction phase, three doses of CombiG-Vax were administered biweekly, followed by a maintenance regimen of monthly booster doses to sustain immune activation [46]. Immune monitoring revealed positive delayed-type hypersensitivity (DTH) responses in a subset of patients, correlating with enhanced progression-free survival (PFS) and overall survival (OS) [46]. Median PFS reached 11.3 months from leukapheresis and 12.2 months from surgery, while the median OS extended to 23.4 months—demonstrating the vaccine’s potential to improve outcomes when integrated with standard therapies [46]. Importantly, the co-administration of temozolomide (TMZ) synergistically enhanced vaccine efficacy, potentially by reducing the immunosuppressive effects of GBM [46]. Notably, no severe treatment-related adverse events were reported, underscoring the vaccine’s favorable safety profile.

The findings from this trial reinforce the potential of personalized DC-based vaccines to overcome GBM’s inherent immunosuppressive barriers. Future directions should explore refining vaccine protocols, assessing the T cell inhibitory molecules to maximize the vaccine’s efficacy and applicability by improving patient selection criteria, and integrating additional immunotherapeutic agents.

### 1.8. DCVax-L—Phase III Trial

The Phase III DCVax-L trial represents a significant milestone in glioblastoma multiforme (GBM) immunotherapy, conducted at 94 sites across the US, Canada, Germany, and the UK [47]. This personalized therapeutic approach leverages autologous dendritic cells (DCs) as professional antigen-presenting cells (APCs) to elicit anti-tumor immune responses. Tumor lysates derived from surgical resections provide patient-specific antigens, addressing GBM’s high genetic and phenotypic heterogeneity. Patients underwent standard-of-care (SOC) surgical resection and chemoradiotherapy before enrollment. Randomization (2:1) stratified patients into SOC plus DCVax-L (n = 232) or SOC plus placebo (PBMCs; n = 99). The DCVax-L vaccine was administered intradermally at predefined intervals, integrating seamlessly with monthly temozolomide (TMZ) treatments to maximize immune activation.

The preparation process for DCVax-L involved a series of stringent steps to ensure its effectiveness and consistency. Tumor tissue resected during surgery (approximately 2 g) was processed into lysates, which were then used to load autologous DCs. Peripheral blood mononuclear cells (PBMCs) were collected via leukapheresis and differentiated into immature DCs using granulocyte-macrophage colony-stimulating factor (GM-CSF) and interleukin-4 (IL-4). A cytokine maturation cocktail, including IL-1β, TNF-α, and IL-6, enhanced the DC phenotype, ensuring robust antigen presentation. The vaccine was aliquoted into doses, cryopreserved at <150 °C, and stored centrally for shipment to trial sites. Each dose contained 2.5 million tumor lysate-pulsed DCs, administered intradermally in alternating arms. The schedule included treatments on Days 0, 10, and 20, followed by Months 2, 4, and 8, and every 6 months from Month 12.

DCVax-L showcased a strong safety profile and significant survival benefits. Mechanistically, the vaccine activated cytotoxic T lymphocytes (CTLs) through antigen presentation by major histocompatibility complex (MHC) molecules, while delayed-type hypersensitivity (DTH) testing confirmed robust T cell responses. Immunological analysis revealed increased CD8^+^ T cell infiltration and reduced regulatory T cells (Tregs) in the tumor microenvironment, favoring anti-tumor immunity. Median progression-free survival (PFS) was 11.3 months from leukapheresis and 12.2 months from surgery, while median overall survival (OS) reached 23.4 months, a substantial improvement over historical benchmarks. Notably, 13% of newly diagnosed patients remained alive at five years compared to 5.7% in the placebo group. The trial’s findings underscore the potential of DC-based vaccines like DCVax-L in addressing glioblastoma’s immunosuppressive barriers. The combination of DCVax-L with SOC therapies demonstrated synergistic effects, enhancing immune responses and improving survival outcomes. These results pave the way for further refinements in vaccine protocols and the exploration of combinatorial strategies to enhance therapeutic efficacy in GBM management.

## 2. Discussion

The past five years have seen significant advancements in the clinical development of cell-based vaccines for glioblastoma multiforme (GBM). These therapies aim to harness the immune system to target and eliminate tumor cells while addressing the unique challenges posed by the immunosuppressive tumor microenvironment (TME) and the blood–brain barrier (BBB) [22,23]. Dendritic cell (DC)-based vaccines remain the most extensively studied approach, as demonstrated by the range of trials summarized in Table 1. These vaccines leverage the antigen-presenting capabilities of DCs to activate cytotoxic T lymphocytes (CTLs) against tumor-associated antigens (TAAs) [18,19,20]. Several studies, including Phase I to Phase III trials, have explored various formulations, antigen-loading strategies, and combinatorial regimens to improve clinical efficacy while maintaining safety profiles [35,40,41,42,43,44,45,46,47].

GBM presents an exceptionally complex landscape due to its highly immunosuppressive microenvironment. The tumor actively suppresses immune activation through mechanisms such as the secretion of immunosuppressive cytokines (e.g., IL-10, TGF-β), the recruitment of regulatory T cells (Tregs) and myeloid-derived suppressor cells (MDSCs), and the overexpression of immune checkpoint molecules like PD-L1 [24,25,26]. Many recent vaccine trials, such as DCVax-L and CombiG-Vax, have shown that DC vaccines can shift the balance of the TME toward an immunostimulatory phenotype, increasing CD8^+^ T cell infiltration while reducing Tregs and MDSCs [46,47]. These effects are crucial in overcoming the tumor’s ability to evade immune responses and prolong patient survival.

One key mechanistic advancement has been the incorporation of immune adjuvants and maturation stimuli, such as poly-ICLC in the GBM6-AD vaccine or keyhole limpet hemocyanin (KLH) in CombiG-Vax, which enhance DC maturation and antigen presentation [35,46]. Additionally, the α-type-1 dendritic cell (DC) vaccine optimized IL-12 secretion to promote a strong Th1-skewed immune response, leading to long-term immune memory formation and a favorable survival benefit [43]. These modifications illustrate a shift toward improving the immunogenicity and durability of vaccine-induced responses.

The integration of DC-based vaccines with the standard of care (SOC) has been another critical area of investigation. Trials such as DCVax-L and ICT-107 have combined autologous DC vaccines with temozolomide (TMZ) chemotherapy, which has been hypothesized to modulate the immunosuppressive TME and enhance vaccine efficacy [42,47]. DCVax-L, the most extensive trial to date, demonstrated a median overall survival (OS) of 23.4 months compared to historical controls of approximately 15 months, highlighting the potential of combining DC-based immunotherapy with SOC [47]. Similarly, CombiG-Vax demonstrated a significant improvement in both progression-free survival (PFS) and OS, underscoring the role of personalized antigen-loading strategies in tailoring immune responses to heterogeneous tumor antigens [46].

However, despite these promising results, some trials have shown limitations in achieving robust survival benefits across all patient populations. For example, the ICT-107 trial found that only specific patient subgroups, such as HLA-A2+ patients with MGMT promoter methylation, benefited significantly from the vaccine [42]. These findings underscore the importance of patient stratification and biomarker-driven selection in future vaccine development. The AV-GBM-1 trial also demonstrated a modest survival benefit, with a median OS of 16 months, suggesting that targeting tumor-initiating cells alone may not be sufficient to overcome GBM’s immunosuppressive barriers [28].

## 3. Future Directions and Combinatorial Strategies

To enhance the clinical impact of cell-based vaccines for GBM, future studies should focus on refining vaccine design, optimizing patient selection, and exploring combinatorial treatment strategies. One promising approach is the integration of checkpoint inhibitors, such as anti-PD-1/PD-L1 antibodies, with DC vaccines to prevent T cell exhaustion and further enhance anti-tumor immunity [13,14]. Additionally, metabolic modulators targeting the immunosuppressive metabolism of GBM cells may provide an avenue for improving vaccine efficacy [16,17]. Another important area of investigation is the role of BBB-penetrating agents in increasing the accessibility of systemic immunotherapies to intracranial tumors [23,24].

Beyond monotherapy approaches, combinatorial strategies incorporating radiotherapy-induced immunogenic cell death (ICD) and oncolytic viral therapy may further enhance the effectiveness of DC-based vaccines [15,16]. For instance, the neoadjuvant vaccination strategy employed in the GBM6-AD trial demonstrated that preoperative vaccination could prime the immune system more effectively than postoperative approaches alone, leading to greater CD8^+^ T cell infiltration into the tumor microenvironment [35]. Such approaches highlight the potential of sequencing and timing in vaccine administration to maximize therapeutic benefit.

## 4. Conclusions

The past five years of research on cell-based vaccines for GBM have yielded significant insights into immunological mechanisms, clinical efficacy, and the integration of immunotherapies with SOC. Trials such as DCVax-L and CombiG-Vax have provided strong evidence supporting the use of personalized DC-based immunotherapy in extending survival for GBM patients, albeit with varying degrees of success across different subgroups. The challenges posed by the immunosuppressive microenvironment, tumor heterogeneity, and BBB remain substantial, necessitating further refinements in vaccine formulations, patient stratification criteria, and combination therapy approaches. The advancement of GBM immunotherapy depends on the incorporation of multimodal treatment regimens, such as immune checkpoint modulation, metabolic alterations, and cutting-edge antigen-loading methods, to improve the effectiveness and clinical outcomes of cell-based vaccines.

## Figures and Tables

**Table 1:** Summary of recent clinical trials on cell-based vaccines for glioblastoma multiforme (GBM): efficacy, preparation, and outcomes.

No.	Vaccine	Trial Phase	Patient Population	Antigen	Company	Preparation	Mechanism of Action	Clinical Outcomes	Key Findings
1	GBM6-AD [35]	Phase I	Low-grade gliomas	Tumor-cell lysate with poly-ICLC	Academic	Tumor-cell lysate combined with poly-ICLC; DCs pulsed with lysate	Priming of the immune system using tumor lysate and adjuvant (poly-ICLC) to activate T cells	Median PFS: 11 months; Median EFS: 23.7 months; enhanced CD8^+^ T cell activity	Neoadjuvant vaccination boosts immune response; need for further optimization
2	Autologous DC Vaccine (Stem-like Lysate) [40]	Phase I	Newly diagnosed or recurrent GBM	Allogeneic stem-like cell line lysate	Academic	DCs pulsed with allogeneic stem-like cell line lysate; matured with IFN-γ and LPS	Activation of T cells by presenting diverse tumor-associated antigens	Median PFS: 8.75 months (newly diagnosed); Median OS: 20.26 months (newly diagnosed)	Promising outcomes in newly diagnosed patients; potential survival benefit
3	DC Vaccine + Gliadel Wafer [41]	Phase I	Malignant gliomas (GBM, AA, AO)	Autologous tumor lysate	Academic	DCs pulsed with autologous tumor lysate; combined with Gliadel Wafers (BCNU)	Localized chemotherapy with BCNU combined with immune activation from DCs	Median OS: 16.9 months; Median PFS: 3.6 months; 25% immune response	Combination approach well tolerated but modest immune response; potential for combinatory therapies
4	ICT-107 [42]	Phase II	Newly diagnosed GBM	MAGE-1, HER-2, AIM-2, TRP-2, gp100, IL13Ra2	ImmunoCellular Therapeutics	DCs pulsed with 6 GBM-associated antigens (MAGE-1, HER-2, AIM-2, TRP-2, gp100, IL13Ra2)	Presents tumor antigens to T cells to target tumor cells and cancer stem cells	Improved PFS in HLA-A2+ patients with MGMT promoter methylation; no significant improvement in OS	Potential benefits for specific subgroups; importance of personalized therapy
5	AV-GBM-1 [28]	Phase II	Newly diagnosed GBM	Tumor-initiating cells	AIVITA Biomedical	DCs pulsed with tumor-initiating cell lysate	Targeting tumor-initiating cells to stimulate a potent immune response	Median PFS: 10.4 months; Median OS: 16.0 months; generally well tolerated	Targeting TICs shows modest survival advantage; safety requires further study
6	α-type-1 DC Vaccine [43]	Phase II	High-grade gliomas including GBM	Glioma-specific peptides	Academic	DCs polarized to α-type-1 state; loaded with glioma-specific peptides	Presents tumor-specific antigens via MHC to activate CTLs; enhances Th1-type response	Median OS: 19 months; median RFS: 11 months; well tolerated	Long-term immune memory induced by IL-12 and IFN-γ interactions; promising for high-grade gliomas
7	CombiG-Vax [46]	Phase II	Newly diagnosed GBM	Autologous tumor lysate	Academic	DCs pulsed with autologous tumor lysate, matured using cytokine cocktail and KLH adjuvant	Antigen presentation to CTLs; reduction in Tregs; increased CD8^+^ T cell activation	Median OS: 23.4 months; Median PFS: 11.3 months	Showed promising immune responses, was well tolerated, and enhanced survival outcomes
8	DCVax-L [47]	Phase III	Newly diagnosed GBM	Autologous tumor lysate	Northwest Biotherapeutics	PBMCs differentiated to DCs, pulsed with tumor lysate, matured, and cryopreserved	Antigen presentation by DCs to prime CTLs; induces anti-tumor immune response	Median OS: 23.4 months; Median PFS: 11.3 months	Demonstrated safety and efficacy in a large-scale trial; significant survival improvement
